# A Comparison of Toxicity Mechanisms of Cigarette Smoke on Isolated Mitochondria Obtained from Rat Liver and Skin

**Published:** 2015

**Authors:** Parvaneh Naserzadeh, Mir-Jamal Hosseini, Sepideh Arbabi, Jalal Pourahmad

**Affiliations:** a *Department of Pharmaceutical Sciences, Islamic Azad University, Tehran, Iran. *; b *Department of Pharmacology and Toxicology, School of Pharmacy, Zanjan University of Medical Sciences, Zanjan, Iran. *; c *Faculty of Pharmacy, Shahid Beheshti University of Medical Sciences, Tehran, Iran.*

**Keywords:** Cigarette smoke extracts (CSE), Toxicity, Isolated mitochondria, Liver, Skin

## Abstract

Previous studies demonstrated that CSE induces oxidative stress and its consequences on isolated mitochondria obtained from lung, heart and brain which may provide insight into the role of CSE in human health and disease. The present study was carried out to further characterize and compare toxic effect of CSE extract on isolated mitochondria obtained from either a directly contacting tissue (*i.e*. skin) or a vital visceral tissue (*i.e*. liver).We obtained Rat liver and skin mitochondria by differential ultracentrifugation and incubated the isolated mitochondria with different concentrations (1, 10 and 100%) ofstandardizedcigarette smoke extract (CSE). Our results were similar to our previous study which discovered CSE toxicity mechanisms on isolated mitochondria obtained from lung, heart and brain with minor changes.CSE induced a significant rise in ROS formation, lipid peroxidation and mitochondrial membrane potential collapse and mitochondrial swelling on isolated mitochondria obtained from both liver and skin. CSE induced Decrease in ATP concentration on isolated mitochondria obtained from both liver and skin did not include CSE lowest concentration (1%). Our findingsshowed that CSE-induced toxicity in liver and skin is due to disruptive effect on mitochondrial respiratory chain which canleads to cytochrome c release and apoptosis signaling.

## Introduction

Cigarette smoking is a complex mixture of 40 different compoundswithtoxic and/or carcinogenic potential (1). Numerous studies showed the potential hazard of cigarette smoke for infants and children (2). High incidence of respiratory tract diseases and cancer in heavy smokers may reflect cigarette smoking induced impairment in the immune system (3). Controversially; cigarette smoke (CS) negatively affects on heart diseases, atherosclerosis, fatty liver diseases and premature skin aging (4-6). Other study suggested that CS caused an imbalance in connective tissue matrix components (7) According to some epidemiological studies; Cigarette smoking in low dose in chronic time induces considerable teratogenic and carcinogenic effects by nicotine on new born rat (8).

Mitochondria are dynamicorganelles essential for cellular life, death, and differentiation. They are best known forATP production via oxidative phosphorylation (OXPHOS), and are centers for apoptosis and ion homeostasis(9,10). Also mitochondrial respiratory chain is a rich source of reactive oxygen species and the cellular production of hydrogen peroxideand they are also vulnerable to oxidative stress (11). Previous studies also showed that CS increases ROS generation inside and outside of mitochondrial respiratory chain. Reactive oxygen species are promoters of chemical modification and conformational changes in membrane polypeptides and lipids (3). Numerous studies have demonstrated that oxidative stress due to the mitochondrial dysfunction plays a key role in tissue injury and cell apoptosis (12). Therefore, we planned to study and compare thetoxicity mechanisms of CS extract on isolated rat mitochondria obtained from a directly contacted peripheral organ (skin) and also a visceral indirectly contacted organ (liver).

## Experimental

*Chemicals*

All chemicals were purchased from Sigma-Aldrich (Taufkrichen, Germany). All chemicals were of the best commercial grade. Cigarette smoke extract (CSE) was standardized and used at lower concentrations (1, 10, and 100%) by diluting 100% CSE in RPMI 1640 with 10% FBS. 

*Animals*

Male Sprague-Dawley rats (200-300 g) that had access ad libitum to water were used in the experiments in a controlled temperature (22 ± 1 °C) and humidity of 70-80% under artificial light with 12 h light/dark cycle. All the experiments were carried out according to established ethical standards approved by the Committee of Animal Experimentation in Shahid Beheshti University of Medical Sciences, Tehan, Iran

*Preparation of mitochondria*

Rats were decapitated and the liver and skinwere surgically harvested, minced and homogenized with a glass hand held homogenizer with previous method (13). Protein concentration was determined by the Coomassie blue protein-binding method using BSA as the standard sample (14). 

*In-vitro* *evaluation of mitochondrial parameters*

The mitochondrial ROS production was assayed by F-2500 fluorescence spectrophotometer (HITACHI) using DCFH-DA in the period of 60 min (15). The activity of mitochondrial complex II (succinate dehydrogenase) was determined by measuring thereduction of MTT (16). The content of the lipid peroxidation marker (MDA) was assessed by measuring the absorbance of the supernatant at 532 nm with an ELISA reader as described in previous study (17). Reduced glutathione (GSH) level was determined in mitochondrial extracts using DTNB reagent using by spectrophotometer. GSH content was expressed as µg/mg protein (18). Mitochondrial membrane potential was determined by mitochondrial uptake of rhodamine 123 with fluorescence spectrophotometer at the excitation and emission wavelength of 490 nm and 535 nm, respectively (19). Mitochondrial swelling was assayed using a previously reported method by monitoring the absorbance at 540 nm (20). The ATP level and ATP/ADP ratio were measured by luciferase enzyme (21). Finally, concentration of cytochrome cwas determined by using the Quantikine^®^ Rat/Mouse Cytochrome cImmunoassay kit (Minneapolis, Minn).

*Statistical analysis*

All experiments were performed with triplicates (N=3). All results are expressed as mean ± SD. Probability p-values <0.05 were considered statically significant.

## Results

As shown in Table 1, CSE concentrations (10 and 100%) induced a significant rise at ROS formation on both liver and skin mitochondria. However, lower concentration of CSE (1%) did not significantly increase mitochondrial ROS generation during 60 min of exposure, compared to control skin mitochondria (P>0.05). Increased ROS formation at each concentration of CSE is expressed as DCF fluorescence intensity unit (Table 1). As shown in Table 2, 1 h exposure of liver and skin mitochondria to different concentrations of CSE (1, 10 and 100%) results in significant decrease in the mitochondrial reduction of MTT to formazan (p<0.05).

**Table 1:** Aqueous cigarette smoke extract (CSE) induced ROS formation on isolated skin and liver mitochondria.

ROS					Groups
60 min	45 min	30 min	15 min	5 5min	
Skin					
29±2	20±2	10±3	2±1	0±1	Control
59±8	44±7	34±5	18±4	3±1	+CSE (1%)
154±13***	141±9***	129±11***	125±7***	23±5***	+CSE (10%)
292±23***	284±14***	266±18***	255±14***	29±5***	+ CSE (100%)
Liver					
14±2	9±2	4±3	2±1	0±1	Control
29±3*	24±5*	21±6*	18±1**	3±2	+CSE (1%)
59±9**	56±9***	51±12***	49±6***	14±4*	+CSE (10%)
88±18***	80±11***	79±8***	75±9***	23±7***	+ CSE (100%)

ROS formation was determined by fluorescence spectrophotometer using DCFH-DA as described in materials and methods and demonstrated as DCF fluorescence intensity unit. Values represented as mean±SD (n=3).

* *P*<0.05;

** *P*<0.01;

*** *P*<0.001 compared with control mitochondria at the same time interval.

**Table 2:** Effect of aqueous cigarette smoke extract (CSE) on Succinate dehydrogenase (complex II) activity (%) on both liver and skin mitochondria.

Succinate dehydrogenase (complex II) activity (%)		Groups
Skin	Liver	
100±1	100±1.400±1.4	Control
82.08±6.3*	85±4.77*	+CSE (1%)
66.06±7.8**	74.9±0.90*	+CSE (10%)
44.45±1.9***	42.50±0.42***	+ CSE (100%)

Succinate dehydrogenase activity was measured using MTT dye as described in Materials and methods. Isolated mitochondria (0.5 mg/mL) were incubated for 1 h with various concentrations of CSE (0, 1, 10 and 100%). Values represented as mean}SD (n=3).

* *P*<0.05;

** *P*<0.01;

*** *P*<0.001 compared with control mitochondria.

On the other hand, addition of concentrations of CSE (10 and 100%) to bothliver and skin mitochondria, significantly increased MDA formation compared to their correspondingcontrol mitochondria. However, lower concentration of CSE (1%) did not significantly increase MDA formation on isolated skin mitochondria (P>0.05)(Table 3).

**Table 3:** Effect of aqueous cigarette smoke extract (CSE) on lipid peroxidationon both liver and skin mitochondria

MDA(μg/mg protein)		Groups
skin	Liver	
3.89±1.54	4.82±1.82	Control
7.94±1.28	8.05±1.97	+CSE (1%)
13.82±0.93**	15.77±0.96**	+CSE (10%)
17.14±1.07***	27.30±0.86***	+ CSE (100%)

Isolated mitochondria (0.5 mg/mL) were incubated for 1h with various concentrations of aqueous CSE(0,1 ,10 and 100%) .Values represented as mean±SD (n=3).

* *P*<0.05 compared with control mitochondria.

Incubation of different CSE concentrations (1, 10 and 100%) significantly decreased GSH levels on isolated mitochondrial obtained from both skin and liver tissues following 1 h compared to their correspondingcontrol mitochondria (P<0.05) (Table 4).

**Table 4:** Effect of aqueous cigarette smoke extract (CSE) on the GSH level on both liver and skin mitochondria.

GSH(μg/mg protein)		Groups
Skin	Liver	
13.68±0.48	54.07±0.50	Control
9.07±1.06***	37.96±1.01**	+CSE (1%)
7.43±1.10***	29.67±0.78***	+CSE (10%)
4.27±0.94***	24.67±0.49***	+ CSE (100%)

Isolated mitochondria (0.5 mg/mL) were incubated for 1h with various concentrations of aqueous CSE (0,1 ,10 and 100%) .Values represented as mean±SD (n=3).

* *P*<0.05 compared with control mitochondria.

The uptake of the cationic fluorescent dye, rhodamine 123, has been used for the measurement of mitochondrial membrane potential collapse. As shown in Table 5, CSE concentrations (1, 10 and 100%) significantly induced MMP collapse onisolated liver mitochondrial after 30 min of incubation (p<0.05) (Table 5). As shown in Table 5, CSE concentration (1%) did not induce significant MMP collapse after 60 min of incubation.

**Table 5:** Effect of aqueous cigarette smoke extract on mitochondrial membrane. Potential MMP collapse (ΔΨ%) on both liver and skin mitochondria.

∆Ψ%					Groups
60 min	45 min	30 min	15 min	5 5min	
Skin					
					
21±2	21±4	17±5	13±1	0±2	Control
41±5**	35±8	26±4	21±5	4±1*	+CSE (1%)
58±4***	55±11*	48±7***	42±16	13±1***	+CSE (10%)
66±7***	61±13**	59±2***	57±18*	22±1***	+ CSE (100%)
Liver					
45±2	31±1	21±5	14±2	0±1	Control
147±11***	86±8***	58±3**	17±4	1±1	+CSE (1%)
149±15***	94±9***	63±9***	22±5	5±2	+CSE (10%)
138±13***	97±7***	86±10***	22±3	6±3	+ CSE (100%)

Mitochondrial membrane potential collapse (**∆**Ψ%) was measured by Rhodamine 123 as described in Materials and Methods. The effect of aqueous CSE concentration% (0, 1, 10 and 100) on the mitochondrial membrane potential decrease on liver and skin mitochondria were evaluated. The values are expressed as means ± SD (n=3). Values represented as mean±SD (n=3).

* P<0.05;

** *P*<0.01;

*** *P*<0.001 compared with control mitochondria.

A decreased light absorbance is consistent with an increase in mitochondrial volume reflected the opening of mitochondrial ion channels and membrane pores.Our result showed that there were a significant decrease in absorbance following incubation of both rat liver and skin mitochondria with different CSE concentrations (1, 10 and 100%) after 45 min of incubation on isolated liver mitochondria and after 1 hour of incubation onisolated mitochondria which is consistent with our MMP collapse and lipid peroxidation results (Table 6). 

**Table 6:** Effect of aqueous cigarette smoke extract (CSE) on the mitochondrial swellingon both liver and skin mitochondria.

Mitochondrial Swelling percent (%)					Groups
60 min	45 min	30 min	15 min	5 min	
Skin					
					
21±2	21±4	17±5	13±1	0±2	Control
41±5**	35±8	26±4	21±5	4±1	+CSE (1%)
58±4***	55±11*	48±7***	42±16	3±1	+CSE (10%)
66±7***	61±13**	59±2***	57±18*	4±3	+ CSE (100%)
Liver					
5±2	3±1	2±1	1±1	0±1	Control
17±2***	16±1***	13±4	11±2*	6±2	+CSE (1%)
41±2***	40±2***	40±4**	30±4***	29±2***	+CSE (10%)
73±1***	72±4***	71±15***	69±4***	69±9***	+ CSE (100%)

Mitochondrial swelling was measured by determination of absorbance at 540 nm as described in Materials and methods. Values represented as mean±SD (n=3).

* P<0.05;

** *P*<0.01;

*** *P*<0.001 compared with control mitochondria.

Wealso measured the ATP levels on isolated mitochondria obtained from rat liver and skin following the addition of CSE concentrations (1, 10 and 100%). As shown in Table 7, CSE concentrations (10 and 100%) significantly decreased mitochondrial ATP levels onbothskin and liver mitochondria compared to their corresponding control mitochondria.ATP depletion is an indicator of mitochondrial dysfunction (Table 7). 

**Table 7:** Effect of aqueous cigarette smoke extract (CSE) on mitochondrial ATP levelon both liver and skin mitochondria.

ATP (µmol/mg protin )		Groups
Skin	Liver	
2.78±0.20	2.61±0.12	Control
2.73±0.19	2.19±0.04	+CSE (1%)
1.28±0.01**	1.72±0.29*	+CSE (10%)
0.89±0.18***	0.64±0.06***	+ CSE (100%)

Isolated mitochondria (0.5 mg/mL) were incubated with CSE% concentrations (0,1,10 and 100) and ATP levels were determined after 1 h of incubation using *Luciferin/Luciferase *Enzyme System as described in Materials and methods. Values represented as mean±SD (n=3).

** *P*<0.01;

*** *P*<0.001 compared with control mitochondria.

Finally, cytochrome c release, important endpoint of cell death signaling was determined. Our results showed thatsignificant(P<0.05) cytochrome c releasefollowing exposure of isolated liver mitochondria to different concentrations of CSE in a concentration dependent manner (Table8),whileonly higher concentrations of CSE (10 and 100%) induced significant (P<0.05) release of cytochrome c from skinmitochondria. Significantly, the pretreatment of CSE-treated mitochondria with MPT inhibitor of cyclosporine A (Cs A) and buthylated hydroxyl toluene (BHT), an antioxidant, inhibited cytochrome c release as compared with CSE-treated group (10%), indicating the role of oxidative stress and MPT pore opening in cytochrome c release following cigarette smoke exposure in both liver and skin tissues(Table 8). 

**Table 8:** Effect of aqueous cigarette smoke extract (CSE) on cytochrome c release on both liver and skin mitochondria.

Cytochrome C release ( ng/mg protein )		Groups
Skin	Liver	
42±17	42±11	Control
50±24	88±17**	+CSE (1%)
101±20*	152±30***	+CSE (10%)
166±8***	254±23***	+ CSE (100%)
84±5	92±43	+CSE (10%) +BHT
80±4	98±40	+CSE (10%) +CsA

Isolated mitochondria (0.5 mg/mL) were incubated for 1h with various concentrations of aqueous CSE (0,1 ,10 and 100%).The amount of released cytochrome c from mitochondria was determined after 1 h of incubation using Rat/Mouse Cytochrome c ELISA kit as described in Materials. Values represented as mean±SD (n=3).

* *P*<0.05 compared with control mitochondria.

## Discussion

According to previous studies, CSE shows liver pathogenesis, including decreased cellular antioxidant levels, increased lipid peroxidation, and increased CYP2E1 induction (22). Besides, fatty liver disease induced by cigarette smokeis associated with cardiovascular disease risk (23). Numerous studies showedCSE causedROS generation via interaction with mitochondrial respiration which could be associated with pathological conditions such as aging, diabetes and cancers (24,25).We thereforeinvestigated and compared toxicity mechanisms of CSE on isolated mitochondria obtained from ratskin and liver.

Based on our results, CSE at various concentrations induced increased ROS formation on both skin and liver mitochondria (Table 1). Mitochondria are an important source of ROS formation in mammalian cells (26). Furthermore, our results showed that decreased complex II(succinate dehydrogenase) activity is involved in CSE-induced tissue damage in both rat skin and liver (Table 2). Based on these results the IC_50_ values for CSE on skin and liver mitochondria were 14.44%, and 45.76% respectively. This suggests that the skin tissue is much more sensitive than liver tissue against CSE toxicity.

Lipid peroxidation has been proven as a major mechanism of free radicals induced cell damage. It may alter intrinsic membrane properties, due to physicochemical changes of oxidized lipids (27). Our results also showed that there was significantMMP collapseon both skin and liver mitochondria after treating with variousconcentrations of CSE. It seems that oxidation of mitochondrial lipid membranes could resultin disruption of mitochondrial membrane potentialand MPT pore opening and finally cytochrome c release. Besides, MPT plays a key role in necrotic celldeath via oxidative stressincluding increasing ROS formation, lipid peroxidation and GSH oxidation (28).

Oxidation of thiol groups (GSH) on both mitochondrial outer or inner membranes could cause conformational change in mitochondrial permeability transition pore (MPT) and also MMP collapse, which are generally considered as potential end points in many conditions associated with oxidative stress (17). Moreover, Cs A and BHT pretreatment completely blocked the CSE-induced release of cytochrome c from both liver and skin mitochondria which supports the hypothesis that the apoptosis induction via CSE is due to an oxidative stress and depends on the opening of the mitochondrial transition pore in liver and skin tissues.Our results confirmed the hypothesis that impairment of ETC by cigarette smoke results in reduced ability of mitochondria for ATP synthesis leading to MPT pores opening which is associated with substantial mitochondrial swelling and finally cytochrome c release on mitochondria obtained from rat liver and skin. 

Based on the IC_50_ values (succinate dehydrogenase activity assay) for CSE on skin and liver mitochondria, the skin tissue is much more sensitive than liver tissue against CSE toxicity. On the other hand, as shown in Table 8, CSE could induce more cytochrome c release and apoptosis signaling in rat liver tissue than skin tissue, perhaps in the latter it favored mostly necrotic mode of cell death. 
